# Effects of Air Plasma Modification on Aramid Fiber Surface and Its Composite Interface and Mechanical Properties

**DOI:** 10.3390/polym14224892

**Published:** 2022-11-13

**Authors:** Ting Xu, Zehao Qi, Qin Yin, Yumin Jiao, Lizhou An, Yefa Tan

**Affiliations:** 1 College of Field Engineering, Army Engineering University of PLA, Nanjing 210007, China; 2 Institute of Defense Engineering, Academy of Military Science of PLA, Beijing 100036, China; 3 94789 Troop of PLA, Nanjing 210018, China

**Keywords:** atmospheric pressure air plasma, treatment time, discharge power, aramid fiber, epoxy resin, interface and mechanical properties

## Abstract

In order to improve the interface and mechanical properties of aramid fiber (AF)-reinforced epoxy resin (EP) composites (AF/EPs), the surface modification of AF was carried out with atmospheric pressure air plasma, and the effects of plasma treatment time and discharge power on the AF surface and the interface and mechanical properties of AF/EPs were investigated. The results show that, when plasma treatment time was 10 min and discharge power was 400 W, AF showed the best modification effect. Compared to the unmodified material, the total content of active groups on the surface of AF increased by 82.4%; the contact angle between AF and EP decreased by 20%; the interfacial energy and work of adhesion increased by 77.1% and 19.1%, respectively; the loss of AF monofilament tensile strength was controlled at only 8.6%; and the interlaminar shear strength and tensile strength of AF/EPs increased by 45.5% and 10.4%, respectively. The improvement in AF/EP interfacial and mechanical properties is due to the introduction of more active groups on the AF surface with suitable plasma processing parameters, which strengthens the chemical bonding between the AF and EP matrix. At the same time, plasma treatment effectively increases the surface roughness of AF, and the mechanical meshing effect between the AF and EP matrix is improved. The synergistic effect of chemical bonding and mechanical meshing improves the wettability and interfacial bonding strength between the AF and EP matrix, which enables the load to be transferred from the resin to the fiber more efficiently, thereby improving the mechanical properties of the AF/EP.

## 1. Introduction

In recent years, the world’s main high-performance fibers, and advanced composites reinforced by these high-performance fibers, have developed rapidly. For example, carbon fiber is used as a load-bearing structure in many engineering fields due to its excellent mechanical properties, fatigue resistance and corrosion resistance. However, the oxidation of carbon fiber at high temperature will lead to its decreased fire resistance. Li [1] conducted in-depth experimental and simulation research on the evolution of mechanical properties of carbon fiber at high temperature. Glass fiber and basalt fiber have excellent high temperature resistance and tensile strength. As part of the reinforcement phase of reinforced concrete or asphalt materials [2,3], they are widely used in construction, road and bridge engineering.

Aramid fiber is a new high-tech synthetic organic fiber composed of aromatic rings and amide bonds on the main chain of macromolecules. As an important national defense material, aramid fiber is widely used in aviation, aerospace, military, transportation and other fields due to its excellent comprehensive properties, such as impact resistance, high strength, high modulus, low density, high temperature resistance, wear resistance and acid and alkali resistance [4,5,6,7]. For example, in aviation, the super-hybrid composite panel formed by aramid fiber and epoxy non-woven fabric and thin aluminum plates through overlapping and hot pressing has extremely high specific modulus and specific strength, and the fatigue life is 100–1000 times that of aluminum alloy plates, meaning it can be used for the fuselage of the aircraft. In aerospace, composite sheets prepared by impregnating aramid fabric with epoxy resin to form aramid prepreg and directly bonding with a honeycomb or foam structure have outstanding impact resistance and can be used to manufacture structural components of spacecraft that can withstand impact forces. In the military field, bulletproof vests and bulletproof helmets made of aramid fibers are not only small in size and light in weight, but also have bulletproof performance that is improved by 40% compared to old-fashioned nylon vests and tank helmets. In the field of transportation, tires made of aramid fiber and rubber compound are light in weight, low in rolling resistance, high in bearing capacity and good in wear resistance, which improves the ride comfort of cars and prolongs the service life of tires. As aramid fiber is widely used, it is called “all-purpose fiber”, and its output and demand are also the largest among synthetic organic fibers.

However, the surface of aramid fibers is relatively smooth, the specific surface area is small, the surface energy is low and the active surface generally does not exceed 10% of the total surface area; it is thus difficult for such fibers to form a solid connection with the matrix through chemical or physical action [8], and the properties of aramid fiber-reinforced composites, especially their mechanical properties, depend to a large extent on the bonding state of the interface between the fibers and the matrix, which limits the wide application of their composites [9,10]. Therefore, the surface modification of aramid fiber has always been a research hotspot. It is an important technical approach towards improving the mechanical properties of aramid fiber-reinforced epoxy composites by using surface modification technology to properly modify the surface of aramid fiber to improve interfacial bonding between the aramid fiber and resin matrix.

At present, the surface modification methods of aramid fibers mainly include chemical modification and physical modification. Chemical modification is to use chemical reagents to chemically react with aramid fibers to introduce polar groups on the surface to increase the surface activity of the fibers, thereby promoting the adhesion between the fibers and the matrix [11]. Although the effect of chemical modification is relatively significant, this method is mostly suitable for processing a small number of fibers and is not suitable for continuous operation; the process of modification treatment is cumbersome and complicated, and the process is difficult to control. Particularly, chemical reagents will generally cause harm and pollution to the human body and the environment. As the mainstream representative of physical modification methods, plasma modification effectively overcomes the shortcomings of chemical modification. Compared to traditional chemical treatment, plasma surface modification is a dry process that is simple and fast with no water or chemical reagents required. It thus has the advantages of energy saving, high efficiency and no pollution, and is a more economical and environmentally friendly treatment technology. A plasma-modified aramid fiber surface is achieved by bombarding the polymer surface with high-energy electrons and ions generated by gas discharge to initiate radical reactions, including cracking, oxidation, radical transfer, disproportionation and coupling reactions on the fiber surface. Under appropriate processing conditions (processing atmosphere, gas flow, processing time, power density, modulation frequency, etc.), the plasma can change the morphology, polarity and structure of the aramid fiber surface, increase the roughness on the fiber surface, introduce polar groups and increase the wetting ability of the specific surface of the fiber, which is conducive to anchoring and infiltration with the resin matrix that thereby improves interface bonding strength between the two phases [12,13].

Gu Ruqian et al. [14] used dielectric barrier discharge to modify the surface of aramid fibers. After different Ar, Ar/O_2_ and Ar/N_2_ atmosphere media blocking plasma treatments, the different effect mechanisms of the fiber surface were explored, and the influence of O_2_ and N_2_ flow on the surface modification effect of aramid fiber was also investigated. The results show that, after the dielectric barrier discharge plasma treatment, the C on the aramid surface decreases, while the O and N increase and new polar functional groups such as -C-O- and -COOH are generated. At the same time, due to the etching effect of plasma, the surface of aramid fiber becomes rough and the bonding ability of aramid fiber and epoxy resin is thus improved. Li Shuang et al. [15] used ammonia gas as the reactive gas when modifying the surface of aramid fiber III with plasma and studied the effect of plasma treatment time and treatment power on the surface properties of the fiber. The results show that the content of -C-N-, -CONH- and other nitrogen-containing polar groups on the surface of aramid III after modification by ammonia plasma increases, the surface roughness increases and the wetting ability is significantly improved. When the gas plasma treatment time is 15 min and the power is 100 W, the interfacial bonding strength of aramid III and epoxy resin increases from 12.9 MPa before treatment to 18.2 MPa, and the contact angle with water decreases from 71.4° before treatment to 46.8°. Liu Zhiying [16] used helium–oxygen atmospheric pressure RF dielectric barrier discharge plasma to modify aramid fabrics and studied the effects of different treatment time, oxygen flow, discharge power and modulation frequency on the structure and properties of aramid fabrics. The results showed that, under different treatment time and oxygen flow conditions, the wettability of the treated aramid fabric was significantly improved, the surface roughness was increased and the tensile strength and flexural strength of the aramid fabric-reinforced composite were significantly improved. Under conditions with different discharge power and modulation frequency, the wettability of the treated aramid fabric first increased and then decreased with the increase in modulation frequency and gradually increased with the increase in discharge power; the surface roughness increased and the tensile and flexural strengths of the material were also significantly improved. However, the above-mentioned plasma modification research has the following problems: (1) The reactive gases used, such as oxygen, argon, nitrogen, helium, etc., all require special equipment and the continuous cost is high; some of them, such as ammonia, also involve toxic gas. (2) It is also necessary to configure expensive and complicated vacuum equipment.

In recent years, atmospheric pressure air plasma treatment has solved the above problems very well, it has thus been widely favored by researchers and become a research hotspot in the field of fiber surface plasma modification. Therefore, this paper uses atmospheric pressure air plasma treatment to modify the surface of AF. By changing the plasma treatment time and discharge power, the effects of these two key process parameters on the aramid fiber surface and the interface properties and mechanical properties of its reinforced epoxy composite are systematically studied. Scanning electron microscope (SEM) and X-ray photoelectron spectroscopy (XPS) were used to analyze the surface morphology and surface element composition of AF. Static contact angle analysis was used to measure the wettability of AF, and its interface energy and work of adhesion were calculated. The tensile strength of AF monofilament, the interlaminar shear strength (ILSS) between AF and EP, and the tensile strength of AF/EP under different plasma process parameters were tested, and the failure mode and reinforcement mechanism of the composites were studied. The object is to provide technical support for the further research and development and engineering application of aramid fiber-reinforced epoxy composite materials.

## 2. Experiment

### 2.1. Materials

Aramid fiber (AF): DuPont Kevlar-29, with an average diameter of 14 µm; Aramid fiber cloth: Model XL-AF480B-100, made of Kevlar-29 fibers, twill weave, square gram weight 480 g/cm^2^, width 100 cm, Yixing Xinli Weaving Co., Ltd. (Yixing, China); ethyl acetate and deionized water were obtained from Shanghai Aladdin Biochemical Technology Co., Ltd. (Shanghai, China); IN2 infusion epoxy resin, AT300SLOW curing agent (IN2:AT300SLOW = 100:30), Beijing Composite Yigou Technology Co., Ltd. (Beijing, China).

### 2.2. Atmospheric Pressure Air Plasma Treatment of Aramid Fiber

**Figure 1:**
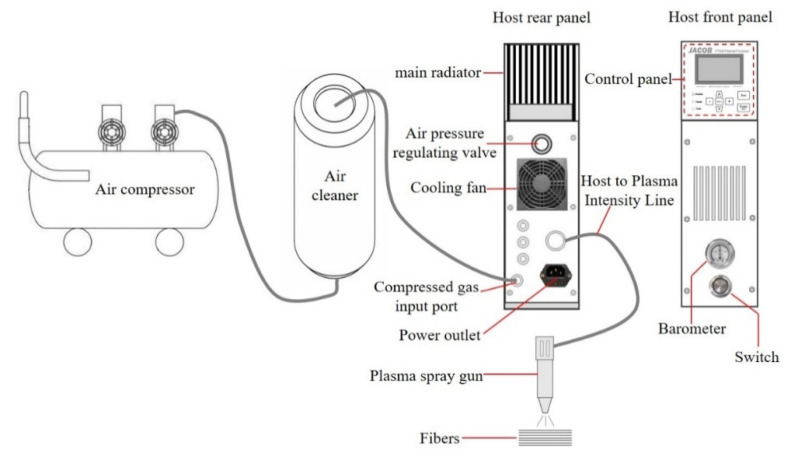
Schematic diagram of the atmospheric pressure air plasma treatment device.

The surface of AF was modified by atmospheric pressure air plasma. The atmospheric pressure air plasma treatment device is manufactured by Nanjing Jiayang Engineering Technology Co., Ltd. (Nanjing, China), model PT800, and its structure and principle are shown in Figure 1. During the test, the key process parameters of treatment time and discharge power were taken as the main research objects. The effects of atmospheric pressure air plasma on the surface modification of AF and the interface and mechanical properties of reinforced epoxy composites under different treatment time (0 min, 5 min, 10 min, 15 min) and different discharge power (0 W, 300 W, 400 W, 500 W) conditions were studied. Other process conditions included room temperature, normal pressure, air as a working gas and a working frequency of 20 KHz.

The technological process and steps are as follows:

(1) Aramid fibers were surface pretreated prior to plasma treatment. First, AF was soaked in ethyl acetate for 24 h for surface pretreatment to remove impurities on the fiber surface, then washed with deionized water 3 times and dried in an oven at 60 °C for 12 h.

(2) The dried aramid fiber was taken out, placed at a distance of 10 mm from the plasma spray gun nozzle, and the plasma spray gun nozzle was moved in the form of a uniform reciprocating movement of 3 cm/s so that the plasma jet at the spray gun mouth could evenly irradiate the fiber surface to ensure the fiber surface treatment effect. Special attention should be paid to not leave the fibers in the plasma jet area for an extended period of time so as to avoid high temperature burns.

### 2.3. Testing and Characterization

The microscopic morphology of the AF surface was observed with a scanning electron microscope (SEM) (Zhongke Keyi KYKY-EM6900), and the samples were sprayed with gold for 2 min before the observation. The acceleration voltage was 25 KV and the magnification of the AF sample was 5000×. Photoelectron spectroscopy (XPS) (ESCALAB 250XI, Waltham, MA, USA) was used to analyze the chemical composition and group composition of the AF surface. An Al Kα X-ray light source was used in the test. The vacuum of the sample chamber was 2 × 10^−8^ Pa. The excitation power and voltage were 40 W and 15 KV, respectively. The Gauss–Lorentz function was used to fit the peaks of C1s spectrum, and the percentage of functional groups on the fiber surface was determined according to the relative peak areas of each peak in the spectrum. Peak C at the binding energy of 284.6 eV was used to correct the displacement.

The static contact angle and surface energy of the AF were measured with a contact angle measuring instrument (DSA100, Kruss, Hamburg, Germany) to characterize the wettability of the AF surface. During the test, a single fiber wire with a length of about 5 cm was cut and bonded to an aluminum hook with adhesive tape. A steel needle was dipped into a small amount of the test liquid and used to gently touch the surface of the single fiber wire to form stable microdroplets. The droplet was photographed with a charge-coupled device and, finally, the static contact angle of the AF was fitting measured by Young–Laplace software.

Based on the GB/T 31290-2014 standard, the tensile strength of the AF monofilament under plasma treatment conditions with different process parameters was tested, and the effect of plasma treatment process parameters on the tensile strength of the AF monofilament was studied. Each AF monofilament was firmly fixed on a paper card frame with adhesive. At room temperature, a 100 N sensor was used for testing, the length of the monofilament was 20 mm and the stretching speed was 1 mm/min. Ten monofilaments were measured in each group.

The aramid fiber-reinforced epoxy resin composites were prepared using the vacuum diversion method. According to the ASTM D2344 standard, the interlaminar shear strength (ILSS) of the composite was measured on the electronic universal testing machine CMT 5105, and the sample was subjected to 3-point bending load. The sample size was 15 mm × 5 mm × 3 mm, the loading speed was 1 mm/min, the span was 12 mm, the test temperature and relative humidity were 25 °C and 50%, respectively, and the average value was taken from 5 samples in each group. The ILSS value of composite materials can be calculated by Formula (1):

(1) $$ILSS=0.75\frac{P_{m}}{b\times h}$$

where the ILSS unit is MPa; $P_{m}$ is the maximum load borne by the specimen at failure, unit N; b is the width of the specimen, unit mm; and h is the thickness of the specimen, unit mm.

The tensile strength of the composite was measured on an electronic universal testing machine (UTM 5504) according to the ASTM D3039 standard. The sample size was 250 mm × 25 mm × 3 mm. In order to prevent slippage or clamping damage, the two ends of the sample were bonded with an aluminum alloy reinforcement sheet (50 mm × 25 mm × 2 mm), the tensile rate was 5 mm/min, the test temperature and relative humidity were 25 °C and 50% and the average value was taken for each group of 5 samples. The tensile strength of the composite can be calculated by Formula (2):

(2) $$\sigma_{t}=\frac{P^{max}}{A}$$

where $\sigma_{t}$ is the tensile strength of the composite material in MPa; $P^{max}$ is the maximum load of the sample before failure, in N; and A is the cross-sectional area of the sample, unit mm^2^.

Scanning electron microscopy (SEM) (Hitachi S-4800, Hitachi, Tokyo, Japan) was used to observe the microscopic morphology of a cross-section of the tensile specimen of composite materials. Before observation, the specimen was put into an ion sputtering instrument to spray gold for 2 min, and the accelerating voltage was 5 KV.

## 3. Results and Discussion

### 3.1. Aramid Fiber Surface Morphology

**Figure 2:**
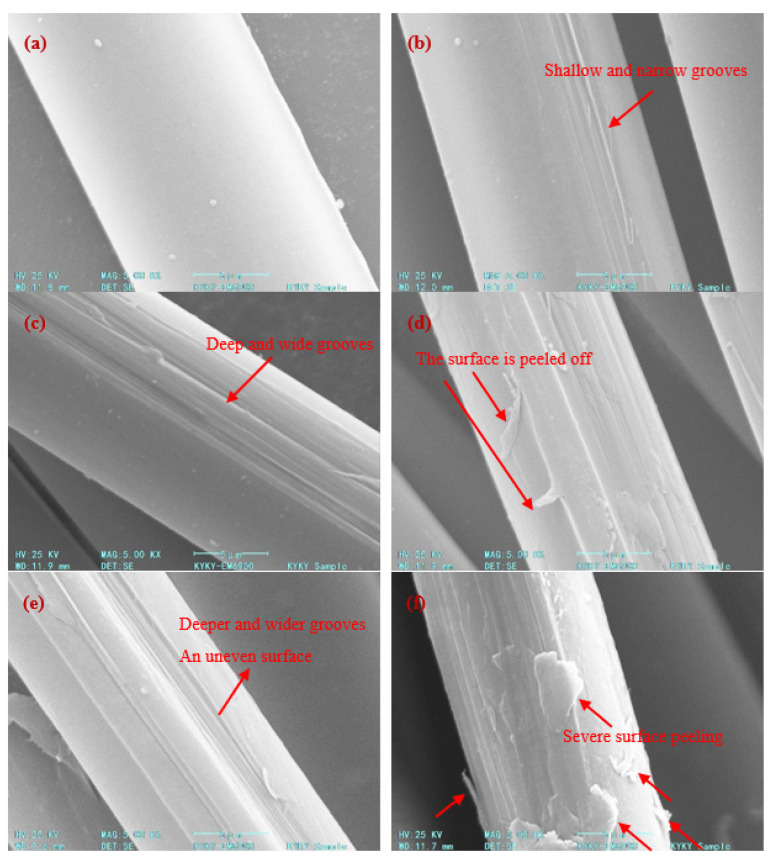
Microscopic surface morphology of AF. (**a**) 0 min, 0 W; (**b**) 5 min, 300 W; (**c**) 10 min, 300 W; (**d**) 15 min, 300 W; (**e**) 10 min, 400 W; (**f**) 10 min, 500 W.

Figure 2 shows the SEM topography of the AF surface under different plasma modification process parameters. It can be seen that the surface of the untreated AF (0 min, 0 W) is very smooth and flat, and no groove structure can be observed. There are several small, raised particles that are likely impurities adhered to the surface (Figure 2a). After treatment, uneven morphology such as grooves and peeling appeared on the surface of the fiber, and the treatment time and discharge power had an important impact on the surface morphology of the fiber (Figure 2b–f).

Under certain discharge power conditions (300 W), when the treatment time was short (5 min), slight etching marks appeared on the surface of the AF, and grooves distributed parallel to the fiber axis were formed on the surface; however, the number of grooves was relatively small, the depth was relatively shallow, the width was relatively narrow and the etching effect was relatively weak (Figure 2b). When the treatment time was extended to 10 min, the etching effect of the AF surface had a further increasing trend (Figure 2c): the number of grooves on the AF surface increased significantly, and the grooves were significantly deepened and widened. At the same time, it can be observed that, in addition to most of the rough areas on the AF surface, there were some smooth areas, which is due to the fact that the aramid fibers wrapped together to form fiber bundles which were placed in the discharge reaction zone; it was thus impossible for the plasma to completely and uniformly contact and treat the fibers, so some fibers in the fiber bundle may only be partially processed. However, if the treatment time is too long (15 min), the etching degree of the AF surface is significantly further increased, with convex edges and deeper grooves, forming an uneven morphology. In particular, there are traces of partial severe peeling and damage on the fiber surface (indicated by the red arrow in Figure 2d).

Comparing Figure 2c,e,f, it can be seen that discharge power has a more significant effect on AF surface morphology. Under the condition that treatment time is fixed at 10 min, when the discharge power is increased from 300 W (Figure 2c) to 400 W (Figure 2e), the surface of AF is etched with more, deeper and wider grooves, and the degree of surface unevenness is more significant, which greatly increases the specific surface area and surface roughness of aramid fibers. The change in the surface morphology of aramid fibers is mainly caused by the rearrangement of the surface’s microscopic molecules and the movement of small molecules under the plasma atmosphere [17]. When a large number of excited particles in the plasma bombard the surface of the fiber continuously, it will cause chain cleavage and cross-linking on the surface of the fiber, thereby roughening the surface of the fiber, increasing the specific surface area and generating polar groups on the surface [18]. However, when the discharge power is too high (500 W), the etching effect on the surface of the fiber is extremely strong, and a large area of serious surface peeling occurs on the surface of the fiber (indicated by the red arrow in Figure 2f). At the same time, the severe etching also causes the surface of the crystalline region inside part of the fiber to be exposed again. It can be speculated that the etching effect of the plasma may have touched the interior of the fiber, and the main structure of the fiber may have been damaged.

Therefore, the plasma treatment has an etching effect on the surface of the aramid fiber, such that the surface roughness of the fiber is significantly increased. Moreover, with increases in plasma treatment time and discharge power, the degree of etching and roughness on the fiber surface both increased gradually. Without damaging the fiber body structure, when the plasma treatment conditions are 10 min-400 W, the fiber surface can obtain greater roughness, which helps to improve the wettability and the interfacial adhesion between the fiber and the matrix and ultimately improves the mechanical properties of the composite. However, it should be noted that when the treatment time is too long (15 min, 300 W) or the discharge power is too large (10 min, 500 W), the fiber body structure will be damaged due to severe surface peeling, which may lead to the deterioration of the fiber and its composite’s material properties.

### 3.2. Chemical Composition of Aramid Fiber Surface

**Figure 3:**
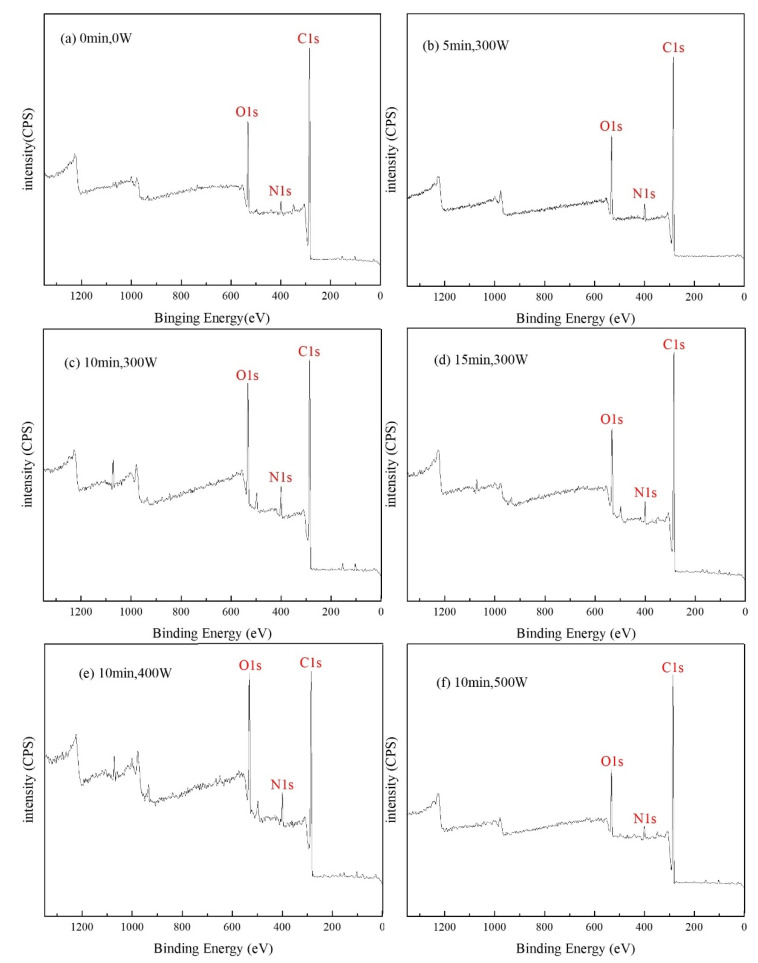
Full XPS spectrum of AF under different plasma treatment process parameters. (**a**) 0 min, 0 W; (**b**) 5 min, 300 W; (**c**) 10 min, 300 W; (**d**) 15 min, 300 W; (**e**) 10 min, 400 W; (**f**) 10 min, 500 W.

Figure 3 shows the full XPS spectrum of AF under different plasma treatment process parameters, and the corresponding element content values are listed in Table 1. The results show that the fiber surface is mainly composed of C, O and N, which is consistent with the constituent elements of the aramid fiber molecular chain. After plasma treatment, the content of C, O and N elements on the surface of treated aramid fibers was significantly different from those of untreated ones and changed with plasma treatment time and discharge power.

The untreated AF surface had C content of 82.63%, O content of 13.8%, N content of 3.57%, an O/C ratio of 0.167 and N/C ratio of 0.043 (Figure 3a). Under the same discharge power conditions (300 W) and with the prolongation of plasma treatment time (5 min, 10 min, 15 min), the C content on the AF surface first gradually decreased and then increased, while the O content and N content both increased gradually and then decreased. When the treatment time was 5 min (Figure 3b), the C content decreased to 80.11%, while the O content and N content increased to 15.47% and 4.42%, respectively. At this time, the O/C ratio and N/C ratio were increased to 0.193 and 0.055, respectively, which was an increase of about 15.6% and 27.9%, respectively, compared to the untreated state. When the treatment time was 10 min (Figure 3c), the C content was further reduced to 74.49%, and the O and N content were further increased to 18.77% and 6.75%, respectively. At this time, the O/C ratio and N/C ratio reached 0.252 and 0.091, respectively, demonstrating a great improvement of about 51% and 112%, respectively, compared to the untreated AF samples. However, when the treatment time was further extended to 15 min (Figure 3d), the C content on the AF surface increased to 81.54%, while the O content and N content decreased to 12.79% and 5.67%, respectively. At this time, the O/C ratio and N/C ratio dropped to 0.157 and 0.070, respectively, which was 37.7% and 23.1% lower than the ratio recorded at 10 min.

Under the condition that treatment time is 10 min, with the increase in discharge power (300 W, 400 W, 500 W) the element content on the AF surface shows the same change rule as the above treatment time; the C content first decreases and then increases, while the O and N content both first increase and then decrease. Among them, when the discharge power was 400 W (Figure 3e), the C content on the AF surface decreased to the lowest recorded value (71.96%), while the O content (20.47%) and N content (7.58%) both increased to the highest. At this time, the O/C ratio and N/C ratio were as high as 0.284 and 0.105, respectively, which was a further improvement of about 13% and 15%, respectively, compared to 300 W and about 70% and 144%, respectively, compared to the untreated AF samples. However, when discharge power was further increased to 500 W (Figure 3f), the C content on the AF surface increased significantly to 84.95%, while the O content (11.02%) and N content (4.03%) both decreased significantly. At this time, the O/C ratio and N/C ratio dropped to 0.130 and 0.047, respectively, which was 54.2% and 55.2% lower than at 400 W. This is because plasma with too high of a discharge power (10 min, 500 W) or with the above-mentioned long treatment time (15 min, 300 W) has a very strong etching or ablation effect, which will cause serious peeling damage on the fiber surface (Figure 2d,f have been analyzed and confirmed) such that the polar groups formed on the fiber surface will be destroyed and reduced [19].

It can be seen from the above analysis that the changes in the relative content of C, O and N fully prove that the plasma treatment has indeed produced a modification effect on the surface of the aramid fiber. In particular, under the appropriate plasma treatment process parameters (10 min, 400 W), O and N content on the AF surface increased significantly, which indicates that a large number of oxygen- and nitrogen-containing functional groups may be introduced into the AF surface. The introduction of these polar functional groups can improve the polarity and chemical activity of the fiber surface, help to improve the wettability of the fiber surface and enhance chemical bonding between the fiber and the matrix.

**Figure 4:**
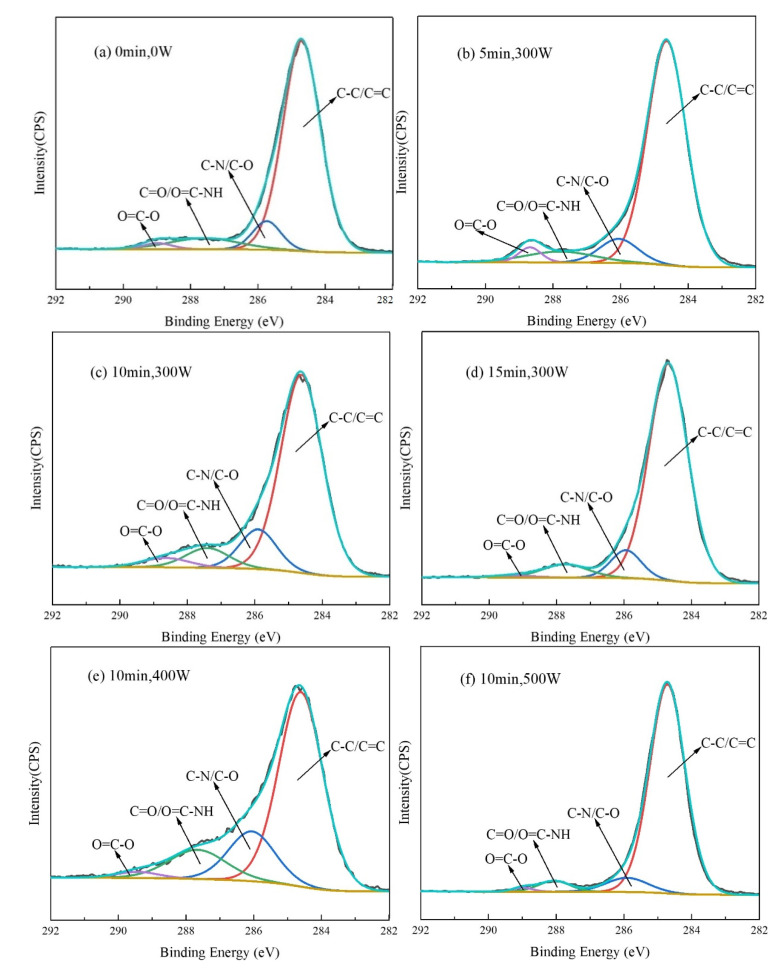
C1s peak spectra of AF under different plasma treatment process parameters. (**a**) 0 min, 0 W; (**b**) 5 min, 300 W; (**c**) 10 min, 300 W; (**d**) 15 min, 300 W; (**e**) 10 min, 400 W; (**f**) 10 min, 500 W.

In order to further explore the effects of plasma treatment time and discharge power on AF surface functional groups, advantage software was used to perform peak fitting processing on the sub-spectra under different plasma treatment process parameters, and the fitting results are shown in Figure 4. Referring to the literature [16,20,21] and combining the structural characteristics of aramid fibers, the spectral peaks around 284.7 eV, 286.3 eV, 287.7 eV and 289 eV correspond to C-C/C=C, C-O/C-N, C=O/O=C-NH and O=C-O groups, respectively. The group composition and content of the AF surface obtained by peak fitting are listed in Table 2.

The results showed that the content of C-C/C=C groups on the surface of untreated AF (Figure 4a) was 81.97%, and the content of polar functional groups C-O/C-N, C=O/O=C-NH and O=C-O was 8.20%, 8.20% and 1.63%, respectively; the total content of polar groups was 18.03%. After plasma treatment, the functional group content on the AF surface changed significantly.

Under the condition of a certain discharge power (300 W) and when the treatment time was short (5 min) (Figure 4b), when compared to untreated AF, the content of C-C/C=C groups on the surface of AF was slightly reduced to 80.65%. At the same time, the content of other polar groups also increased slightly to 19.35%. In general, however, the group content on the fiber surface changed less due to the shorter treatment time. When the treatment time increased to 10 min (Figure 4c), compared with 5 min, the content of C-C/C=C groups on the AF surface was further significantly reduced to 75.19%, and the content of C-O/C-N groups increased significantly from 8.87% at 5 min to 13.53% at 10 min, with the content of C=O/O=C-NH and O=C-O groups also increasing slightly compared with 5 min. At this time, the total content of polar groups was 24.81%, an increase of 28.2% compared with 5 min treatment time and an increase of 37.6% compared with untreated materials, indicating that the proportion of polar groups on the AF surface continued to increase. However, when the treatment time was further extended to 15 min (Figure 4d), the content of C-C/C=C groups on the AF surface did not decrease but increased (up to 84.75%), the content of C-O/C-N, C=O/O=C-NH and O=C-O groups decreased significantly from 13.53%, 7.52% and 3.76% at 10 min to 8.47%, 5.93% and 0.85%, respectively, and the total content of polar groups at this time decreased by 38.5% compared to the content at 10 min.

The next consider condition was a treatment time of 10 min with a gradual increase in discharge power (300 W, 400 W, 500 W). Compared to the discharge power of 300 W (Figure 4c), as the discharge power increased to 400 W (Figure 4e), the content of C-C/C=C groups on the AF surface further decreased significantly from 75.19% at 300 W to the lowest value of 67.11%, and the content of C-O/C-N and C=O/O=C-NH groups was further increased from 13.53% and 7.52% at 300 W to 18.12% and 12.75%, respectively. In particular, at this time, the total content of polar groups was the highest with a value of 32.89% (Figure 4e), which was higher than the value at 300 W (24.81%) by 32.6% and a significant increase of 82.4% compared to the total content of polar groups on the untreated AF surface (18.03%). However, when the discharge power was further increased to 500 W (Figure 4f), the content of C-C/C=C groups on the AF surface increased significantly to 86.95% and the content of C-O/C-N, C=O/O=C-NH and O=C-O groups decreased significantly from 18.12%, 12.75% and 2.02% at 400 W to 7.83%, 4.35% and 0.87%, respectively. At this time, the total content of polar groups was 13.05%, which was lower than that of the untreated material (18.03%) by 27.6%.

As mentioned in the analysis, the chemical bonds included in aramid fibers include C-C, C-N, C=O, O=C-O, etc. These chemical bonds can match the energy of electrons, ions, metastable particles, UV-visible light and other particles in plasma. Therefore, in the process of plasma treatment, the particles can cause these chemical bonds to break and recombine to generate new chemical bonds and new functional groups [14,21]. The chemical reaction equation is as follows:

Reaction with atomic oxygen:

RH+2O^●^→R^●^+H^●^+O_2_

R_1_R_2_+O^●^→R_1_^●^+R_2_O^●^

RH+O^●^→R^●^+O^●^H

Reacts with molecular oxygen:

R^●^+O_2_→ROO^●^

Reacts with peroxide free radicals:

ROO^●^+RH→ROOH+R^●^

R^●^+2ROOH→ROO^●^+R-O^●^+H_2_O

$$R^{\prime }-O^{●}+\mathrm{RH}\to R^{\prime }-\mathrm{OH}+R^{●}$$

According to the above reaction, the principle of modifying an aramid fiber surface by plasma treatment is mainly reflected in two aspects. On the one hand, the plasma bombardment of the aramid fiber surface produces a large number of free radicals and triggers a chain reaction of free radicals. N and O are implanted into the aramid surface, and a large number of oxygen-containing groups are introduced, such as carbonyl, carboxyl and hydroxyl groups. On the other hand, the plasma also etched the AF surface and increased the surface roughness of the fiber. The plasma modification mechanism is actually the combined effect of these two factors. For the aramid fiber samples treated with 15 min, 300 W (Figure 4d) and 10 min, 500 W (Figure 4f) plasma, the content of C-C/C=C groups on the surface increased and the proportion of active functional groups on the surface decreased greatly. This is because plasma treatment for too long a time or of too high a power etches the surface of the aramid fiber excessively, destroys the polar groups introduced on the surface of the fiber in the initial stage of modification, and may even cause the carbonization phenomenon due to local ablation of the fiber surface [20].

Therefore, the change in group content on the surface of aramid fiber before and after plasma modification is completely consistent with the change in C, O and N element content on the fiber surface discussed above. Under the premise that the modification process and other plasma discharge parameters are determined, treatment time and discharge power have a significant impact on the chemical composition of the fiber surface. Appropriate treatment time and discharge power, such as 10 min and 400 W in this study, can ensure that the content of polar groups introduced on the fiber surface achieves a relatively ideal value, which can greatly improve the chemical inertness of the fiber surface, help the matrix to infiltrate the fiber, and strengthen the chemical bonding and polar effect of the interface between the two phases.

### 3.3. Wetting Properties of Aramid Fiber and Epoxy Resin

**Figure 5:**
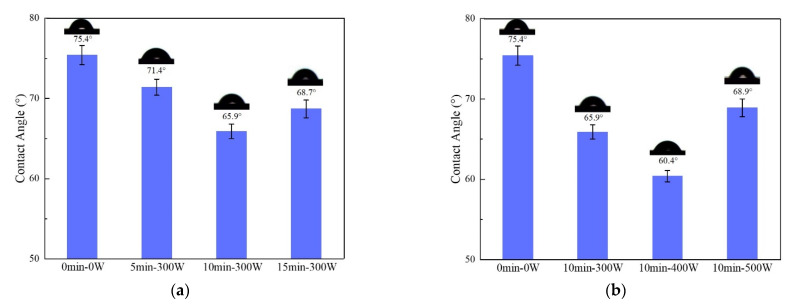
Contact angle of EP droplets on AF (**a**) under different treatment time conditions and (**b**) under different discharge power conditions.

The interfacial and mechanical properties of aramid fiber-reinforced resin matrix composites are closely related to the wettability between fibers and resins [22]. Figure 5 shows the contact angles between AF and EP before and after plasma modification with different treatment times and discharge power. From the test results, the contact angle between untreated AF and EP is 75.4°. After plasma modification, the contact angle changed significantly.

Under the condition of constant discharge power (300 W) (Figure 5a) and with a treatment time of 5 min, the contact angle between AF and EP was 71.4°, which was 5.3% lower than in the untreated state. When the treatment time was extended to 10 min, the contact angle was further reduced to 65.9°, which was 12.6% lower than the untreated state. However, when the treatment time was further extended to 15 min, the contact angle of the two phases increased slightly (68.7°). With a maintained treatment time of 10 min (Figure 5b), discharge power (300 W, 400 W, 500 W) was gradually increased. When discharge power was increased to 400 W, the two-phase contact angle was further reduced compared to at300 W, and it was reduced to the minimum value of 60.4° (which is 20% lower than in the untreated state). However, when discharge power was further increased to 500 W, the contact angle between AF and EP rebounded and rose to 68.9°.

In order to quantitatively analyze the surface energy of AF under different plasma treatment process parameters, the interfacial energy and work of adhesion between AF and EP under different plasma treatment process parameters were calculated according to the Young–Dupre equation [23]. The relationships among AF surface energy, contact angle between AF and EP and work of adhesion between AF and EP are shown in Equations (3) and (4):

(3) $$\gamma_{EA}=\gamma_{A}-\gamma_{E}cos\theta$$

(4) $$W_{EA}=\gamma_{E}+\gamma_{A}-\gamma_{EA}$$

In the above formula, $\gamma_{A}$ is the surface energy of AF; $\gamma_{E}$ is the surface energy of EP; $\gamma_{EA}$ is the interfacial energy between AF and EP; $W_{EA}$ is the work of adhesion between AF and EP; and θ is the contact angle between AF and EP.

According to the surface energy component method of Owens, D.K.J. and Wendt, R.C.J. [24], two typical liquids, deionized water (highly polar) and diiodomethane (non-polar), were selected to test the contact angles between them and AF, and the surface energy of AF and its polar and non-polar components were calculated by Equations (5)–(7).

(5) $$\gamma_{L}\left(1+cos\theta \right)=2\sqrt{\gamma_{A}^{P}\gamma_{L}^{P}}+2\sqrt{\gamma_{A}^{d}\gamma_{L}^{d}}$$

(6) $$\gamma_{A}=\gamma_{A}^{P}+\gamma_{A}^{d}$$

(7) $$\gamma_{L}=\gamma_{L}^{P}+\gamma_{L}^{d}$$

In the above formula, $\gamma_{A}$ is AF surface energy; $\gamma_{L}$ is the test liquid’s surface energy; $\gamma_{A}^{P}$ is the AF surface energy polar component; $\gamma_{A}^{d}$ is the AF surface energy non-polar component; $\gamma_{L}^{P}$ is the polar component of the test liquid’s surface energy; $\gamma_{L}^{d}$ is the non-polar component of the test liquid’s surface energy; and θ is the contact angle between the AF and the test liquid. Among them, $\gamma_{L}^{d}$ and $\gamma_{L}^{P}$ of the deionized water are 21.8 mJ/m^2^ and 51 mJ/m^2^, respectively. $\gamma_{L}^{d}$ and $\gamma_{L}^{P}$ of diiodomethane are 50.8 mJ/m^2^ and 0 mJ/m^2^, respectively.

**Figure 6:**
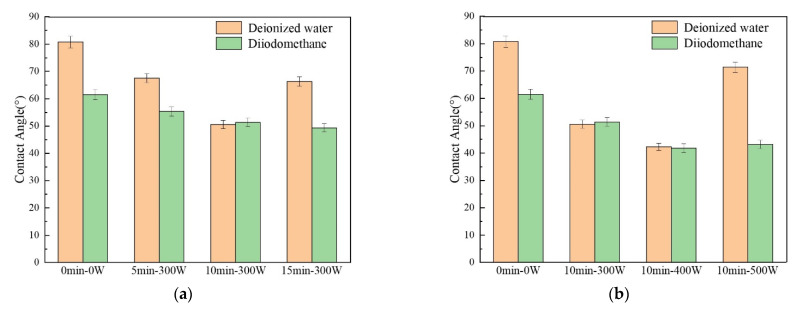
Contact angles between deionized water, diiodomethane and AF (**a**) under different treatment time conditions and (**b**) under different discharge power conditions.

The contact angles between AF and deionized water and diiodomethane under different plasma treatment process parameters are shown in Figure 6, and the corresponding specific values are shown in Table 3. From the test results, from the AF sample without plasma treatment to the samples treated for 5 min, 10 min and 15 min (with discharge power fixed at 300 W) (Figure 6a), the contact angle between AF and water was 80.8°, 67.5°, 50.6° and 66.3°, showing that with the prolongation of the plasma treatment time, the contact angle gradually decreased at first and then increased significantly. Among them, the AF surface treated for 10 min at 300 W had the minimum contact angle (50.6°). Under the same treatment time (10 min), the effect of discharge power on the contact angle of AF (Figure 6b) and the effect of treatment time were roughly the same, that is, as the discharge power increased from 300 W to 500 W, the contact angle of AF and water first decreased and then increased; it first decreased to a minimum value of 42.3° (10 min, 400 W), and then significantly increased to 71.4° (10 min, 500 W). After plasma treatment, the contact angle of AF and diiodomethane was reduced (by about 10–32%) as a whole compared with to without treatment, and kept fluctuating between 55.3° and 41.8° with a minimum value of 41.8° at 10 min and 400 W.

**Figure 7:**
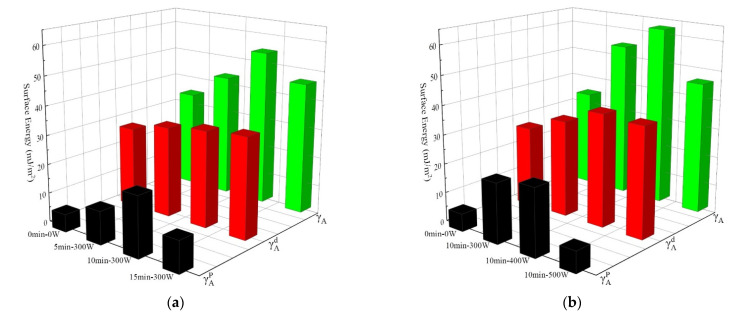
Surface energy of AF under different plasma treatment process parameters: (**a**) different treatment time conditions; (**b**) different discharge power conditions. (Black represents the AF surface energy polar component $\gamma_{A}^{P}$, Red represents the AF surface energy non-polar component $\gamma_{A}^{d}$, Green represents the AF total surface energy $\gamma_{A}$).

According to formulas (5)–(7), the change law of the surface energy of AF can be calculated from the contact angles of AF with deionized water and diiodomethane, as shown in Figure 7, and the corresponding values are listed in Table 3. It can be seen that the surface energy polar component ($\gamma_{A}^{P}$) of the untreated AF is 6.1 mJ/m^2^. Under the condition of a discharge power of 300 W (Figure 7a) and with the prolongation of treatment time, $\gamma_{A}^{P}$ first gradually increased and then decreased. $\gamma_{A}^{P}$ increased to 11.4 mJ/m^2^ after 5 min treatment, and $\gamma_{A}^{P}$ further significantly increased to 20.6 mJ/m^2^ after 10 min treatment; however, when treatment time was extended to 15 min, $\gamma_{A}^{P}$ decreased to 10.8 mJ/m^2^. At the same time, when treatment time was 10 min (Figure 7b) with a gradual increase in discharge power (300 W, 400 W, 500 W), $\gamma_{A}^{P}$ showed the same change rule. It first gradually increased to the maximum value of 23.0 mJ/m^2^ at 400 W and then reduced to 7.3 mJ/m^2^ at 500 W. The surface energy non-polar component ($\gamma_{A}^{d}$) increased after plasma modification as a whole compared to untreated material, and reached a maximum value of 38.9 mJ/m^2^ when treated for 10 min at 400 W. The change rule of AF total surface energy ($\gamma_{A}$) is consistent with its polar component ($\gamma_{A}^{P}$). $\gamma_{A}$ was 33.9 mJ/m^2^ for untreated material. Under the condition of a discharge power of 300 W (Figure 7a), $\gamma_{A}$ increased to 42.7 mJ/m^2^ after 5 min of treatment, and $\gamma_{A}$ further increased to 53.9 mJ/m^2^ after 10 min of treatment; however, after a longer (15 min) plasma treatment, $\gamma_{A}$ dropped to 45.4 mJ/m^2^ again. At the same time, with 10 min treatment time (Figure 7b) and an increase in discharge power, $\gamma_{A}$ reached the maximum value of 61.9 mJ/m^2^ at 400 W, an increase of 14.8% compared to 300 W, and an even more substantial increase of 82.6% compared to the untreated material. When discharge power was further increased to 500 W, $\gamma_{A}$ decreased to 45.3 mJ/m^2^.

Through analysis and discussion of the test results for AF static contact angle and the calculation results of AF surface energy, it can be found that: (1) Proper prolongation of treatment time and increases in discharge power can both produce better fiber surface modification effects (AF surface contact angle decreases and surface energy increases). This is because the fibers will be bombarded by active particles more with longer treatment time, and the modified positions will be more uniform. Additionally, increasing discharge power can directly increase the average energy and number of active particles in the plasma, such that the intensity of the plasma modification on the fiber surface increases under the same length of treatment time. (2) There are two reasons for the above changes in the contact angle and surface energy of AF. On the one hand, some new polar groups were generated on the AF surface after plasma modification (confirmed by the XPS test results), thus increasing the polar component of fiber surface energy. On the other hand, as can be calculated from the Wentzel equation, when *θ* < 90°, the increase in surface roughness will decrease the contact angle [25]. The etching effect of the plasma makes the fiber surface rougher (confirmed by the SEM test results), thereby reducing the contact angle of the fiber surface and, at the same time, increasing the non-polar component of fiber surface energy. However, if the treatment time is too long or the discharge power is too high (such as 15 min, 300 W or 10 min, 500 W), it will lead to peeling of the uneven structure of the fiber skin, that is, a new active surface may be generated. This will lead to the aramid surface contact angle increasing. At the same time, the polar functional groups introduced on the fiber surface will be destroyed and removed due to the excessively strong etching effect of the plasma, resulting in a decrease in the surface energy of AF.

The interfacial energy and work of adhesion between the AF and EP under different plasma treatment process parameters were further calculated by Formulas (3) and (4), and quantitative analysis of the interfacial properties between AF and EP was realized. The calculation results are listed in Table 4. Among them, the EP surface energy $\gamma_{E}$ is 41 mJ/m^2^ [26].

It can be seen from Table 4 that, after the plasma modification treatment, the interfacial energy and work of adhesion between AF and EP changed significantly with treatment time and discharge power, and the change rule is consistent with that of AF surface energy $\gamma_{A}$. Among them, when the plasma treatment conditions were 10 min and 400 W, the interfacial energy and work of adhesion between AF and EP reached maximum values (41.8 mJ/m^2^ and 61.1 mJ/m^2^, respectively), representing increases of 77.1% and 19.1%, respectively, compared with the untreated condition.

Therefore, when the treatment time for the atmospheric pressure air plasma modified AF surface is 10 min and the discharge power is 400 W, the surface contact angle of AF is the smallest, the surface energy of the AF is the largest, and the interfacial energy and work of adhesion between AF and EP are the highest. These conditions also allow for the best wettability, which will be beneficial through increases in van der Waals force, enhanced chemical bonding and mechanical locking between EP and AF in the interface region, and a reduced number of defects such as bubbles, holes and cracks existing in the AF/EP composite, thereby improving its interfacial and mechanical properties.

### 3.4. Monofilament Tensile Properties of Aramid Fibers

From the previous SEM observations, it can be seen that the surface morphology of AF changed significantly after plasma treatment: the AF surface is etched and the roughness is also significantly increased. These changes may have some impact on the body properties of the fibers. In order to study the surface modification of AF by plasma in terms of the body properties of the fibers, the fiber monofilament tensile strength test was used to analyze changes in fiber tensile properties before and after plasma modification. In this study, statistical analysis is used to test the experimental results, that is, the Weibull distribution function [27,28,29] is used for statistical analysis of the test data. Thus, more detailed and accurate information on how the body mechanical properties of AF are changed after plasma modification can be obtained.

The one-dimensional two-parameter Weibull distribution function [30] is expressed as Equation (8):

(8) $$F\left(\sigma_{f}\right)=1-exp\left[-L{\left(\frac{\sigma_{f}}{\sigma_{0}}\right)}^{m}\right]$$

In Equation (9), $\sigma_{f}$ represents AF monofilament tensile strength; L is tensile distance; $F\left(\sigma_{f}\right)$ is the failure probability, which, when the tensile distance is *L*, is the cumulative distribution function of fracture probability for AF when the fracture stress is not higher than $\sigma_{f}$; $\sigma_{0}$ is the scale parameter of the Weibull distribution under corresponding tensile distance; and m is the shape parameter of the Weibull distribution under corresponding tensile distance.

Equation (9) with survival probability P can be obtained by deformation of Equation (8):

(9) $$P=1-F\left(\sigma_{f}\right)=exp\left[-L{\left(\frac{\sigma_{f}}{\sigma_{0}}\right)}^{m}\right]$$

Subsequently, taking the natural logarithm of both sides of Equation (9) obtains Equation (10):

(10) $$lnln\left[1/\left(1-F\left(\sigma_{f}\right)\right)\right]=mln\sigma_{f}+lnL-ln\sigma_{0}^{m}$$

It can be seen from Equation (10) that there is a linear relationship between $lnln\left[1/\left(1-F\left(\sigma_{f}\right)\right)\right]$ and $ln\sigma_{f}$, and the least squares method [31] can be used to estimate the Weibull scale parameter $\sigma_{0}$ and the shape parameter m. The specific steps are as follows: the tensile strength of a batch of N single AF monofilaments is measured, and the test results $\sigma_{i}$ are arranged in an increasing sequence from the smallest to the largest: $\sigma_{1}<\sigma_{2}<\cdots <\sigma_{i}<\cdots <\sigma_{N}$.

The failure probability $F\left(\sigma_{f}\right)$ of the material can be calculated by Equation (11):

(11) $$F\left(\sigma_{f}\right)=i/\left(N+1\right)$$

In Equation (11): N is the total number of AF monofilaments tested in each group, in this case it is equal to 10; i is the serial number after sorting the test strength data from smallest to largest, in this article it is 1,2…10, which gives us ten pairs of numbers ($F\left(\sigma_{f}\right),\sigma_{f}$).

By linear distribution of Equation (10), Equations (12)–(15) can be obtained as follows:

(12) $$Y=lnln\left[1/\left(1-F\left(\sigma_{f}\right)\right)\right]$$

(13) A=m

(14) $$X=ln\sigma_{f}$$

(15) $$B=lnL-ln\sigma_{0}^{m}$$

Equation (16) can be obtained by slightly changing Equation (15):

(16) $$\sigma_{0}=exp\left(\left(lnL-B\right)/m\right)$$

Using the least squares method to perform linear regression analysis on the 10 pairs of numbers ($F\left(\sigma_{f}\right),\sigma_{f}$), $lnln\left[1/\left(1-F\left(\sigma_{f}\right)\right)\right]$ is fitted to $ln\sigma_{f}$ to form the line Y=AX+B. According to the fitting results, and by solving Equations (13), (15) and (16), the Weibull parameters m and $\sigma_{0}$ of the material are obtained.

AF average monofilament tensile strength $\bar{\sigma }$ is further calculated by Equation (17):

(17) $$\bar{\sigma }=\sigma_{0}L^{-1/m}\Gamma \left(1+1/m\right)$$

In the above formula, Γ represents the Gamma function.

**Figure 8:**
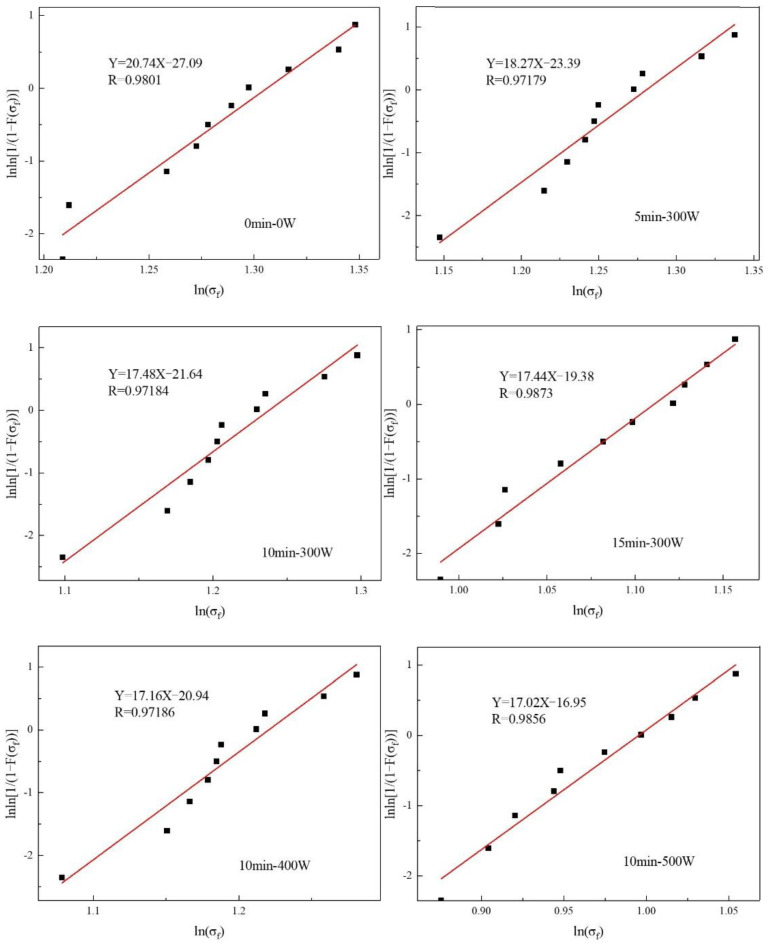
Weibull distribution curve of the tensile strength of AF monofilaments under different plasma treatment process parameters.

The experimental data of each group of 10 AF monofilaments treated under different plasma treatment process parameters were tested, and the tensile distance was 20 mm. Using the Weibull distribution to perform statistics and analysis on the test data, $lnln\left[1/\left(1-F\left(\sigma_{f}\right)\right)\right]$ was taken as the ordinate and $ln\sigma_{f}$ as the abscissa to draw a scatter plot of the Weibull distribution and perform linear fitting (Figure 8). It can be seen that the linear fitting of the test data is good, which indicates that the tested AF monofilament tensile strength data conform to the Weibull distribution theory. According to the intercept and slope of the fitted line, the shape parameter m and the scale parameter $\sigma_{0}$ can then be calculated, as listed in Table 5. In the Weibull analysis, the scale parameter $\sigma_{0}$ characterizes monofilament tensile strength. The larger the scale parameter is, the larger the monofilament tensile strength is. The shape parameter m characterizes the dispersion of the fiber strength distribution. It can be seen that, after the plasma modification treatment, the shape parameter m was reduced compared to that of the untreated AF (m was 20.74), though the overall shape parameter m did not change much during the entire plasma treatment process, as they both remained at about 17–18. This shows that, after plasma modification, the strength dispersion of aramid fiber samples is higher than that of untreated AF fibers, which may be related to an inability to ensure completely uniform plasma discharge. Additionally, different plasma treatment process parameters do not have much influence on the strength distribution of aramid fibers.

**Figure 9:**
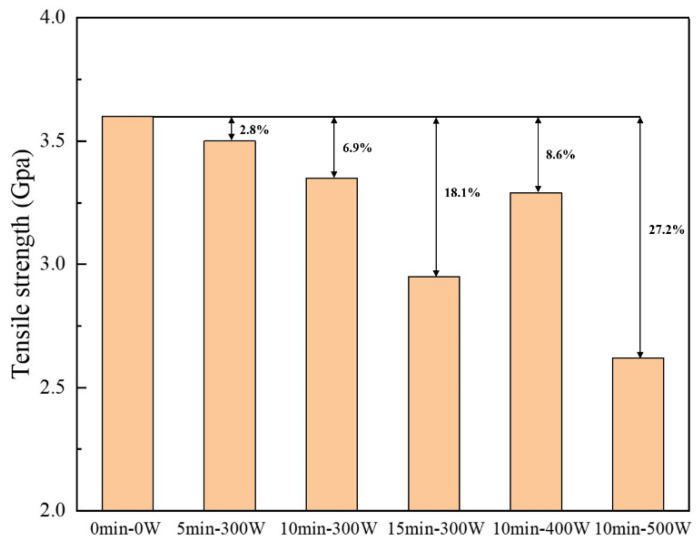
Average single-filament tensile strength of AF under different plasma treatment process parameters.

Through further calculation by Equation (17), the average single-filament tensile strength of AF under different plasma treatment process parameters can be obtained, as shown in Figure 9. It can be seen that the untreated AF monofilament has a tensile strength of 3.60 GPa. After plasma treatment, the average single-filament tensile strength of aramid fibers decreased. When the discharge power was kept at 300 W, and when the treatment time was 5 min and 10 min, the decrease rates were 2.8% and 6.9%, respectively, indicating that the plasma did not cause great damage to the properties of the fiber under these two conditions. It can be considered that its body properties are basically well preserved in these two cases. This is because the treatment of aramid fiber for this length of time only acts at a depth of several nanometers to 100 nanometers in the outermost layer of the fiber [32]. It has no effect on the fiber body while changing the surface morphology of the fiber, and the treatment time is relatively short. Therefore, the strength damage after treatment is small. However, after 15 min of treatment, since the grooves on the fiber surface become deeper and deeper, the degree of fiber etching will gradually increase, which is large enough to damage the body strength of the fiber and result in a substantial decrease in the fiber’s strength (decrease of 18.1% compared to untreated fibers). At the same time, with a treatment time of 10 min and with a discharge power of 400 W, which can significantly modify the surface chemical composition, physical morphology and wettability of AF, the average tensile strength of AF monofilaments drops to 3.29 GPa, which is a decrease of 8.6% compared to the untreated state (the range of decrease was still controlled within 10%). Therefore, it can be considered that the damage to fiber strength is still small. When discharge power was further increased to 500 W, the average tensile strength of AF filaments significantly dropped by 27.2% to 2.62 GPa compared to the untreated state.

From observation of the SEM images of AF above, it can be concluded that after plasma modification of the AF surface, the modification process will etch the AF surface due to chemical reactions and destroy the surface structure of the AF; the bombardment of active particles in the plasma will also introduce some defects on the fiber surface. When the external load acts on the AF surface, it becomes easy to form a stress concentration at the defect such that the AF will break under a small load, resulting in the phenomenon of reduced average monofilament tensile strength in the AF [33]. In particular, when AF is subjected to plasma treatment for too long or at too high a power (such as 15 min-300 W and 10 min-500 W), the defects introduced by excessive plasma etching on the AF surface are relatively more serious and an obvious surface peeling phenomenon even occurs (Figure 2d,f), resulting in damage to the AF’s body structure. Therefore, the decrease in the AF’s average monofilament tensile properties is more obvious.

The test results and analysis of the tensile strength of AF monofilaments show that plasma modification with different process parameters causes different degrees of damage to the tensile properties of the AF’s body. However, under suitable plasma treatment process parameters, such as the 10 min-400 W treatment in this study, this damage can be controlled to within a relatively small range, which can effectively improve the chemical composition and physical morphology of the AF surface and improve the wettability of the AF surface; importantly, the reduced average monofilament tensile strength was controlled at 8.6%. This quality control is very important for improvements to the interfacial and mechanical properties of AF/EP composites.

### 3.5. Interlaminar Shear Strength (ILSS) of Aramid Fiber-Reinforced Epoxy Resin Matrix Composites

**Figure 10:**
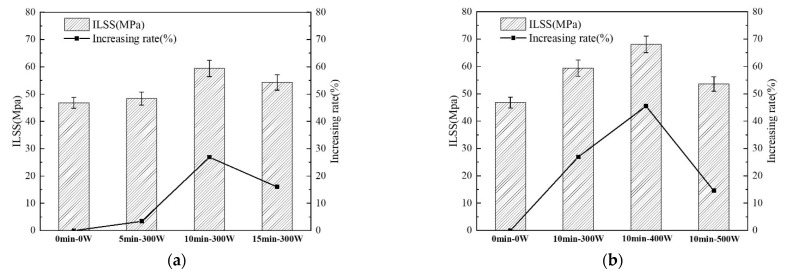
ILSS between AF and EP (**a**) under different treatment time conditions and (**b**) under different discharge power conditions.

In order to analyze and evaluate the interfacial bonding properties of aramid fiber-reinforced epoxy resin composites (AF/EP), the interlaminar shear strength (ILSS) of AF/EP under different plasma treatment times and discharge power was tested according to the ASTM D2344 standard. The test results are shown in Figure 10.

It can be seen from Figure 10 that the ILSS of untreated AF/EP is 46.8 MPa, while after plasma treatment the ILSS of AF/EP was improved in different degrees. (1) The effects of treatment time on the ILSS of AF/EP (Figure 10a) are as follows: With discharge power of 300 W and after plasma treatment for 5 min, the ILSS of AF/EP increased to 48.4 MPa, an increase of 3.4% compared to the untreated condition. By prolonging the treatment time to 10 min, the ILSS of AF/EP was further increased to 59.4 MPa, a significant increase of 26.9% compared to the untreated material. After prolonging the treatment time to 15 min, the ILSS of AF/EP was 54.3 MPa, which is 16% higher than that of untreated AF but lower than that of the 10 min treatment. This shows that plasma treatment for a long time (15 min) has reduced efficiency in improving the interface bonding properties of the AF/EP and a certain negative effect compared with 10 min treatment. (2) The effects of discharge power on the ILSS of AF/EP (Figure 10b) are as follows: From the AF/EP without plasma treatment to the AF/EP treated with 300 W, 400 W and 500 W (with a treatment time of 10 min), the ILSS first gradually increased to the maximum value of 68.1 MPa (400 W) and then decreased to 53.6 MPa (500 W). It can be seen that with prolongation of the treatment time and an increase in discharge power, the change law of the ILSS of AF/EP is basically the same, showing a trend of first increasing and then decreasing. Among them, when the plasma treatment conditions were 10 min and 400 W, the ILSS value of AF/EP was the largest (68.1 MPa), and its growth rate was as high as 45.5% compared to the ILSS value of untreated AF/EP.

The degree of ILSS improvement is affected by the physical structure, roughness, polarity and chemical activity of the AF surface and the ability of the resin to wet the fibers. In particular, under suitable plasma treatment conditions (10 min, 400 W), the ILSS of AF/EP is significantly improved. This is because (1) plasma treatment introduces a large number of polar groups (such as hydroxyl groups, carboxyl groups, etc.) on the surface of the AF, and these polar groups form strong chemical bonds with the resin matrix through a series of reactions; and (2) the etching effect of plasma on the AF surface increases the surface roughness of the fiber, and many grooves and bumps are generated on the fiber surface. These grooves and bumps are equivalent to creating many anchorage points for resin infiltration, which thus increases the contact area between AF and EP and enhances the mechanical chimerism between the two phases. It is precisely under the synergistic effect of chemical bonding and mechanical chimerism that the interfacial bonding strength of AF/EP has been greatly improved. However, plasma treatment for a longer time or with higher power (such as 15 min-300 W or 10 min-500 W) will destroy the active groups formed on the AF surface at the initial stage of plasma treatment such that the content of polar groups on the AF surface is reduced, the chemical bonding between the fibers and the resin is weakened, and the synergistic effect with the mechanical chimerism is weakened, resulting in a decrease in the interface bonding performance of the AF/EP. This also shows that, although the surface roughness of AF and the content of polar groups both have an effect on the interface bonding performance of AF/EP, the content of polar groups on the AF surface has a greater effect on the interface bonding performance of AF/EP than that of AF surface roughness. That is to say, after plasma modification of AF/EP, the contribution of chemical bonding between the AF and EP matrix to improving the interface bonding performance of AF/EP is greater than that of mechanical chimerism.

### 3.6. Tensile Strength ($\sigma_{t}$ ) and Tensile Section Analysis of Aramid Fiber-Reinforced Epoxy Resin Matrix Composites

**Figure 11:**
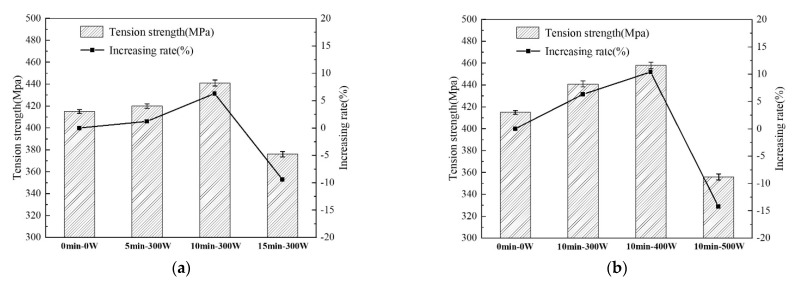
The tensile strength ($\sigma_{t}$) of AF/EP (**a**) under different treatment time conditions and (**b**) under different discharge power conditions.

In order to analyze and evaluate the mechanical properties of aramid fiber-reinforced epoxy resin composites (AF/EP), according to the ASTM D3039 standard, the tensile strength ($\sigma_{t}$) of AF/EP under different plasma treatment times and discharge power was tested. The results are shown in Figure 11.

It can be seen from Figure 11 that the tensile strength of untreated AF/EP is 415 MPa, while after plasma treatment the tensile strength ($\sigma_{t}$) of AF/EP changed significantly. (1) The effects of treatment time on the tensile strength ($\sigma_{t}$) of AF/EP (Figure 11a) are as follows: At a fixed discharge power of 300 W and after plasma treatment for 5 min, the tensile strength ($\sigma_{t}$) of AF/EP is 420 MPa, a slight increase of 1.2% compared to the untreated material; the tensile strength ($\sigma_{t}$) of AF/EP increased to 441 MPa by prolonging the treatment time to 10 min, which is 6.3% higher than the untreated material. However, continuing to prolong the treatment time to 15 min dropped the tensile strength ($\sigma_{t}$) of AF/EP to 376 MPa, which is 9.4% lower than that of the untreated material. (2) The effects of discharge power on the tensile strength ($\sigma_{t}$) of AF/EP (Figure 11b) are as follows: From the AF/EP without plasma treatment to the AF/EP after plasma treatment at 300 W, 400 W and 500 W (with a treatment time of 10 min), tensile strength ($\sigma_{t}$) also shows a trend of increasing first and then decreasing. $\sigma_{t}$ first increased to 458 MPa at 400 W, an increase of 10.4% compared to the untreated material, and the maximum tensile strength value was obtained; however, when discharge power increased to 500 W, tensile strength ($\sigma_{t}$) decreased to 356 MPa, which was 14.2% lower than that of the untreated material.

From the test results, the tensile strength ($\sigma_{t}$) of AF/EP exhibits the following changes: (1) With the appropriate extension of treatment time and the appropriate increase in discharge power, the tensile strength ($\sigma_{t}$) of the AF/EP increased. The reason is that, as discussed above, the chemical elements of the AF surface were greatly changed by the plasma modification (the content of O and N elements increased significantly), and polar groups such as hydroxyl and carboxyl groups increased, such that chemical bonding with the resin was stronger. In addition, the plasma treatment also significantly increased the surface roughness of the AF, which was favorable for the infiltration of the resin and the formation of mechanical locks. It is precisely because of the synergistic enhancement of chemical bonding and mechanical chimerism between AF and EP that the interfacial bonding performance (ILSS) of AF/EP was significantly improved. Therefore, the tensile strength ($\sigma_{t}$) of AF/EP also correspondingly improved and reached the maximum value with 10 min-400 W treatment; this group of optimal process parameters is also consistent with the aforementioned analysis. (2) The maximum increase in the tensile strength ($\sigma_{t}$) of AF/EP was 10.4%, which was observed with the 10 min-400 W treatment because the tensile strength of fiber composites depends on the combined effect of fiber body strength and fiber–matrix interface bonding strength. Although plasma treatment increases interfacial bonding strength, at the same time it also damages the body properties of AF to a certain extent (which was confirmed in the previous monofilament tensile strength test and analysis). The strength of the fiber body also plays an important role in the mechanical properties of the composite material. Therefore, the increase in AF/EP tensile strength ($\sigma_{t}$) is limited. (3) After longer of higher power plasma treatment (15 min-300 W or 10 min-500 W), the tensile strength ($\sigma_{t}$) of AF/EP (376 MPa and 356 MPa, respectively) was lower than that of the untreated material (415 MPa). The reason for this phenomenon is that the excessively violent plasma treatment caused great damage to the body performance of the AF. For example, the average monofilament tensile strength of AF after treatment for 15 min at 300 W was 18.1% lower than that of the untreated material, and the average monofilament tensile strength of AF after treatment for 10 min at 500 W decreased by 27.2% compared to the untreated material. With this treatment time, although the ILSS of AF/EP was still higher than that of the untreated material, when the stress was transmitted from the matrix to the fiber through the interface or when the crack propagation encountered the fiber, the fiber could no longer fully play an effective strengthening role and could no longer prevent crack propagation, thus resulting in a substantial decrease in the overall tensile strength of the composite.

**Figure 12:**
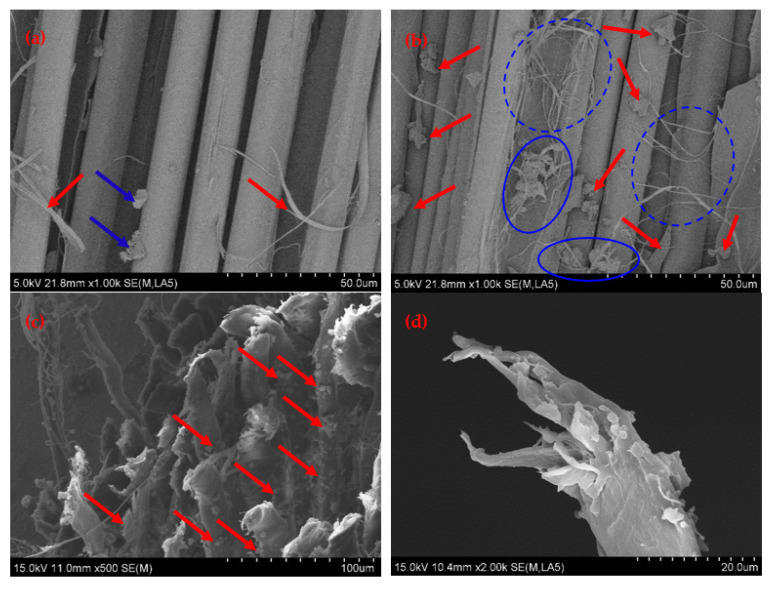
Micromorphology of the tensile section of AF/EP. (**a**) 0 min-0 W (the face parallel to the fiber); (**b**) 10 min-400 W (the face parallel to the fiber); (**c**) 10 min-400 W (the face perpendicular to the fiber); (**d**) 10 min-400 W (single fiber decapitated).

In order to further analyze the reinforcement mechanism and failure mechanism of the fiber composites, the microscopic morphology of the tensile section of AF/EP under 0 min-0 W (untreated) and 10 min-400 W plasma treatment conditions was observed by SEM, as shown in Figure 12. As can be seen from Figure 12a, on the untreated tensile section, the AF and EP matrix are almost completely debonded, and the surface of the pulled fiber is relatively smooth with almost no resin adhesion phenomenon. There are a few small pieces of EP debris on the surface of the fiber (indicated by the blue arrow in Figure 12a), showing typical brittle fracture characteristics. At the same time, a few fiber filaments in the AF fiber bundle after tensile fracture appeared on the fiber surface (indicated by the red arrow in Figure 12a), which means that the fiber was slightly damaged by loading but that the amount of loaded fiber was lower. This is due to poor wettability between AF and EP without plasma treatment (contact angle 75.4°) and lower interfacial energy and work of adhesion between them (23.6 mJ/m^2^ and 51.3 mJ/m^2^, respectively). As a result, interface bonding performance between the AF and EP is poor (ILSS is 46.8 MPa), it is difficult to effectively transfer the load from the resin matrix to the aramid fiber through the interface, and the reinforcing effect and load-bearing effect of the aramid fiber cannot be fully exerted. Therefore, at this time, the tensile strength of AF/EP is relatively low (415 MPa), and only a small load is required to cause an interfacial debonding phenomenon that causes the overall failure of the composite material; its failure mode is mainly an interface debonding failure.

As can be seen from Figure 12b, under the appropriate treatment conditions (10 min, 400 W), a large amount of epoxy resin adhered to the surface of the aramid fiber on the tensile section of AF/EP, and a large amount of resin was filled in between the fibers (the fibers are tightly combined with the resin matrix). The surface of AF/EP also appeared to contain a large amount of epoxy resin block debris (indicated by the red arrow in Figure 12b), with a certain plastic deformation also occurring that is confirmed in Figure 12c (indicated by the red arrow in Figure 12c). At the same time, a large number of fiber filaments and filament-like stripping in the AF fiber bundle after tensile fracture appeared on the fracture surface (the area marked by the blue dotted circle in Figure 12b), and a large number of fiber filaments and plastically deformed EP debris were entangled and intersected (the area marked by the blue solid circle in Figure 12b), which indicates that a large number of fibers were damaged under load and that this was accompanied by a certain plastic deformation of the EP matrix, which fully exerts the load-bearing efficiency of the fibers and the energy absorption effect of the interface matrix. This is because, after modification treatment with proper plasma process parameters (10 min, 400 W), a large number of active groups such as hydroxyl and carboxyl groups were introduced on the surface of the AF, which formed stable covalent bonds between the AF and EP matrix. At the same time, the surface roughness of AF was effectively increased by plasma modification, and the mechanical meshing effect between it and the EP matrix was improved. The synergistic effect of chemical bonding and mechanical meshing significantly improved the interfacial energy (41.8 mJ/m^2^) and work of adhesion (61.1 mJ/m^2^) between the AF and EP, and improved interfacial bonding strength between the AF and EP (ILSS is 68.1 MPa). Additionally, its internal fiber bundles were subjected to greater shear stress when loaded, which results in slippage and then tearing of microfibers of fiber [34] and prompts a certain plastic deformation of the interface matrix to absorb more energy. In addition, it can be seen from the fiber decapitated in Figure 12d that the typical ductile fracture of the aramid fiber occurred with treatment for 10 min at 400 W. When the stress was effectively transferred from the resin matrix to the aramid fiber through the interface, the aramid fiber’s load-bearing capacity was stronger, it could withstand higher stress, and it played an important role in energy absorption, stress relaxation and the strengthening effect. Therefore, AF/EP showed higher tensile strength (458 MPa) when treated with proper plasma parameters (10 min, 400 W).

## 4. Conclusions

In order to improve the interface properties and mechanical properties of aramid fiber-reinforced epoxy resin composites, the surface of AF was modified by atmospheric pressure air plasma and aramid fiber-reinforced epoxy composites were prepared. The effects of the key process parameters (plasma treatment time and discharge power) on aramid fiber surface modification and its reinforced epoxy composite’s interfacial and mechanical properties were investigated.

The results show that when the plasma treatment conditions are 10 min and 400 W, the AF exhibits the best modification and enhancement effect. Compared to untreated material (0 min-0 W), the surface O/C ratio and N/C ratio of AF increased by 70% and 144%, respectively, and the total content of polar groups increased by 82.4%; the contact angle between AF and EP reduced by 20%, the interfacial energy and work of adhesion increased by 77.1% and 19.1%, respectively, and the loss of average single-filament tensile strength was controlled at 8.6%. Additionally, the ILSS and tensile strength of AF/EP were increased by 45.5% and 10.4%, respectively. The improvement in the interfacial and mechanical properties of aramid-reinforced epoxy composites is due to the introduction of more active groups on the surface of the AF through the use of suitable plasma treatment conditions, which strengthens the chemical bonding between the AF and EP matrix. At the same time, the plasma treatment effectively increased the surface roughness of the AF and improved its mechanical engagement with the EP matrix. The synergistic effect of chemical bonding and mechanical meshing makes aramid and epoxy form an organic whole, which significantly improves wettability and interfacial bonding strength between the AF and EP matrix and enables the load to be transferred from the resin to the fiber more effectively. This also allows for more uniform load bearing within the AF, which enables full exertion of its energy absorption, stress relaxation and enhancement effects, thereby improving the mechanical properties of AF/EP.

## Figures and Tables

**Table 1:** Element composition and content for the AF surface under different plasma treatment process parameters.

Sample Code	Chemical Composition (at. (%))			Atomic Ratio	
	C	O	N	O/C	N/C
0 min-0 W	82.63	13.8	3.57	0.167	0.043
5 min-300 W	80.11	15.47	4.42	0.193	0.055
10 min-300 W	74.49	18.77	6.75	0.252	0.091
15 min-300 W	81.54	12.79	5.67	0.157	0.070
10 min-400 W	71.96	20.47	7.58	0.284	0.105
10 min-500 W	84.95	11.02	4.03	0.130	0.047

**Table 2:** Composition and content of surface functional groups for AF under different plasma treatment process parameters.

	Content of Functional Groups (%)			
	C-C/C=C	C-O/C-N	C=O/O=C-NH	O=C-O
0 min-0 W	81.97	8.20	8.20	1.63
5 min-300 W	80.65	8.87	7.26	3.22
10 min-300 W	75.19	13.53	7.52	3.76
15 min-300 W	84.75	8.47	5.93	0.85
10 min-400 W	67.11	18.12	12.75	2.02
10 min-500 W	86.95	7.83	4.35	0.87

**Table 3:** Contact angles and surface energies of deionized water and diiodomethane on AF.

	Contact Angle (°)		Surface Energy (mJ/m^2^)		
	Deionized Water	Diiodomethane	$\gamma_{A}^{P}$	$\gamma_{A}^{d}$	$\gamma_{A}$
0 min-0 W	80.8	61.5	6.1	27.8	33.9
5 min-300 W	67.5	55.3	11.4	31.3	42.7
10 min-300 W	50.6	51.4	20.6	33.3	53.9
15 min-300 W	66.3	49.3	10.8	34.6	45.4
10 min-400 W	42.3	41.8	23.0	38.9	61.9
10 min-500 W	71.4	43.2	7.3	38	45.3

**Table 4:** Interfacial energy and work of adhesion between AF and EP.

	Contact Angle (°)	Interfacial Energy (mJ/m^2^)	Work of Adhesion (mJ/m^2^)
0 min-0 W	75.4	23.6	51.3
5 min-300 W	71.4	29.6	54.1
10 min-300 W	65.9	37.1	57.8
15 min-300 W	68.7	30.6	55.8
10 min-400 W	60.4	41.8	61.1
10 min-500 W	68.9	30.5	55.8

**Table 5:** Weibull distribution parameters of the tensile strength of AF monofilaments under different plasma treatment process parameters.

	Weibull Distribution Parameters			
	B	A=m	R	$\sigma_{0}(\mathrm{GPa})$
0 min-0 W	−27.09	20.74	0.98	4.27
5 min-300 W	−23.39	18.27	0.97	4.24
10 min-300 W	−21.64	17.48	0.97	4.09
15 min-300 W	−19.38	17.44	0.99	3.61
10 min-400 W	−20.94	17.16	0.97	4.03
10 min-500 W	−16.95	17.02	0.99	3.23

## Data Availability

The data presented in this study are available on request from the corresponding author.

## References

[1] Li C., Xian G. (2019). Experimental and Modeling Study of the Evolution of Mechanical Properties of PAN-Based Carbon Fibers at Elevated Temperatures. Materials.

[2] Napoli A., Realfonzo R. (2020). Compressive strength of concrete confined with fabric reinforced cementitious matrix (FRCM): Analytical models. J. COMC.

[3] Fu L., Jiao Y., Chen X. (2022). Reinforcement evaluation of different fibers on fracture resistance of asphalt mixture based on acoustic emission technique. Constr. Build. Mater..

[4] Gao J., Yang X., Huang L.H. (2018). Numerical prediction of mechanical properties of rubber composites reinforced by aramid fiber under large deformation. Compos. Struct..

[5] Ramasamy N., Arumugam V., Suresh Kumar C. (2022). Characterization of fiber/matrix interfacial bonding strength of chemically grafted aramid fiber surface with epoxy resin composites. Polym. Compos..

[6] Cheng Z., Li X., Lv J., Liu Y., Liu X. (2021). Constructing a new tear-resistant skin for aramid fiber to enhance composites interfacial performance based on the interfacial shear stability. Appl. Surf. Sci..

[7] Dharmavarapu P., Reddy M.B.S.S. (2021). Aramid fiber as potential reinforcement for polymer matrix composites: A review. Emerg. Mater..

[8] Hu B.Q., Niu J.C. (2008). Advanced Composite Materials.

[9] Wang Y., Qu R., Pan F., Jia X., Sun C., Ji C., Zhang Y., An K., Mu Y. (2017). Preparation and characterization of thiol- and amino-functionalized polysilsesquioxane coated poly (p-phenylenetherephthal amide) fibers and their adsorption properties towards Hg (II). Chem. Eng. J..

[10] Wang W., Li R., Tian M., Liu L., Zou H., Zhao X., Zhang L. (2013). Surface silverized meta-aramid fibers prepared by bio-inspired polydopamine functionalization. ACS Appl. Mater. Interfaces.

[11] Ling X.L., Zhou Y., Huang J.W., Yue X.X., Jiang F., Lin H.T. (2011). Progress in surface modification of aramid fibers. J. Tianjin Polytech. Univ..

[12] Chen P., Li H., Wang J., Su F. (2008). Study on Surface Modification of High-Performance Organic Fibers by Plasma Technology. Fiber Compos. Mater..

[13] Ling X.L., Guo L.F., Lin H.T. (2016). New progress in the modification of aramid fiber. J. Tianjin Polytech. Univ..

[14] Gu R.Q., Yu J.R., Hu C.C., Chen L., Zhu J., Hu Z.M. (2013). Effects of different plasma atmospheres on surface modification of aramid fiber by dielectric barrier discharge technique. J. Donghua Univ. Nat. Sci..

[15] Li S., Cao Q.Z., Han K.Q., Zhang Y., Yu M.H. (2014). Surface modification of aramid fiber Ⅲ by ammonia plasma treatment. China Synth. Fiber Ind..

[16] Liu Z.Y. (2014). DBD Plasma Treatment of Aramid Fabric and the Mechanical Properties of Its Reinforced Composites. Master’s Thesis.

[17] Xiao W.W. (1992). The Principle and Method of Synthetic Fiber Modification.

[18] Hu F.Z. (2003). Material Surface and Interface.

[19] Wu G.M. (2004). Oxygen plasma treatment of high-performance fibers for composites. Mater. Chem. Phys..

[20] Jia C.X. (2012). The Modification Research of Aramid Surface and Its Reinforced Composite Materials Interface by Air DBD Plasma. Ph.D. Thesis.

[21] Ji J.Y. (2012). Study on the Surface Modification of Aramid Ⅲ and Its Structure and Properties of Epoxy Composite System. Ph.D. Thesis.

[22] Leal A.A., Deitzel J.M., McKnight S.H., Gillespie J.W. (2009). Interfacial behavior of high-performance organic fibers. Polymer.

[23] Schrader M.E. (1995). Young-Dupre Revisited. Langmuir.

[24] Owens D.K., Wendt R.C. (1969). Estimation of the surface free energy of polymers. J. Appl. Polym. Sci..

[25] Jang J., Yang H. (2000). The effect of surface treatment on the performance improvement of caron fiber/polybenzoxazine composites. J. Mater. Sci..

[26] Weldon D.G. (2007). Failure Analysis of Paints and Coatings. Pigm. Resin Technol..

[27] Zhou Y.N., Li W. (2015). WEIBULL analysis and Study on monofilament strength of carbon fiber. High-Tech. Fibers Appl..

[28] Wang C.T., Xie J.F., Qiu Y.P. (2011). WEIBULL theory was used to evaluate the effect of surface treatment on tensile strength of carbon fibers. Ind. Text..

[29] Wang H.W., Wang J.H., Huang Z.M., Zhu J.Y. (2003). WEIBULL was used to evaluate the effect of surface treatment on glass fiber strength. J. Wuhan Univ. Technol..

[30] Sakin R., Ay I. (2008). Statistical analysis of bending fatigue life data using Weibull distribution in glass-fiber reinforced polyester composites. Mater. Design.

[31] Gong J.H., Li Y. (1997). Statistical properties of least squares estimator of WEIBULL modulus for ceramic materials. J. Ceram..

[32] Wang J. (2007). Study on the Interface Properties of Aramid Fibers Modified by Dielectric Barrier Discharge Plasma. Master’s Thesis.

[33] Ren Y., Wang C., Qiu Y. (2008). Aging of surface properties of ultrahigh modulus polyethylene fibers treated with He/O2 atmospheric pressure plasma jet. Surf. Coat. Tech..

[34] Yue M., Zhang C., Du Z.J. (2008). Modification of PBO fiber surface by low temperature plasma. China Synth. Fiber Ind..

